# Indents

**Published:** 1904-11-15

**Authors:** 


### INDENTS.

☞ **Brief Articles of Technical or Scientific Value will receive notice and comment. Contributions invited.**

**Delicacy of Manipulation.** One of the most practical things that can be taught to young operators is the necessity for developing a delicate touch in their manipulations about the mouth. Patients are becoming more and more discriminating about such things, and they rebel against an operator who is unnecessarily rough. The mere examination of the teeth is frequently made a disagreeable, if not painful, procedure through the rude manner in which the lips are forced back and the mirror is jammed about the mouth. A little delicaciy on the part of the operator, particularly at the beginning of any sitting, will help the patient wonderfully and inspire confidence It will also be more highly appreciated than many operators realize.—*Dent. Review.*

**Dangerous To be Safe.** There is danger in being alive these days· If you bathe you are liable to get pneumonia or weaken the constitution. If you don't bathe you will not be able to eliminate the poison from your system that accumulates daily and must be thrown off in some way. Drink beer or water and get typhoid fever. Drink milk and get tuberculosis. Drink whiskey, get jim jams or snakes. Eat soup, get Bright's disease; eat meat, get apoplexy; eat oysters and acquire toxemia; eat vegetable diet and deteriorate or run down the general system. Smoke cigarettes and go crazy. Smoke cigars or a pipe and get a cancer. Drink coffee or tea and get nervous prostration. Drink high wines and get the gout. Now the only way to be perfectly healthy is to eat nothing, drink nothing, smoke nothing. Try it for about six months and then let's hear from you.—I. F. Steele, D. D. S., in *Amer. Journal.*

**The X-Ray for Pyorrhea Alveolaris.** A lady of about forty presented herself for examination. I found the mouth in a very bad condition—teeth loose (I think every one was affected, if I remember correctly); deep pockets, especially on the molars, both upper and lower; left superior central very loose and elongated; gums receded on labial surface almost to pockets on approximal and lingual surfaces; right lower cuspid loose, could have removed it with fingers; pus oozing from all pockets, breath very bad; no appetite; complexion blotched. I first banded the elongated and loose central then ground it off to match the others, removed what deposit I could conveniently, then began exposures to the X-rays. After a few weeks conditions began to improve; the soreness left the teeth and they began to get firmer; pus, which had been so profuse, had almost entirely ceased to flow. They continued to improve. The gum on the central, which had receded, has come down to normal condition and festooned as prettily as it ever was. The pockets, after continued treatment, all closed up. The teeth are all firm, and the gums, as far as I can see, are normal. The lady's complexion is improving very much, and her general health is good, as is also her appetite. I used no drugs of any kind, either locally or internally—nothing but the X-rays, and she is clinically cured. How long she will remain so I cannot say.—Dr. M. L. Schmitt in *Summary.*

**Appeddicitis** Prof. John E. Murphy, in the *American Journal of Medical Science,* for August, makes some valuable deductions from 2,000 operations he has made for appendicitis. Foreign bodies were found in only two percent of the cases. Fecal concretions were found in thirty-eight percent. Bacteria were found in most cases, but he does not regard it as an infectious or contagious disease. It seems to have family predilections. The symptoms in order of occurrence are: Pain in the abdomen, sudden and severe, followed by nausea or vomiting within three or four hours. There is general abdominal sensitiveness, but particularly over the region of the appendix. Elevation of temperature in from two to twenty four hours after pain begins. Any variation of this order indicates some other disorder. The time to operate is first, within the first forty-eight hours; secondly, from the second to the fifth day; thirdly, from the fifth to seventh day and on; fourth, between attacks. The mortality will vary with the skill of the operator, from two percent upward, also according to the period of operating. The majority of deaths are due to not calling the physician early enough, or to mistaken diagnosis. The surgeon who is having more than three or four deaths in a hundred is either receiving his patients from incompetent or procrastinating medical practitioners, or he is doing too much manipulating in the peritoneal cavity under unfavorable pathologic conditions.

**New Occlusal Lines for Artificial Dentures.**

Anatomists tell us that in the normal occlusion of the natural teeth the entire upper arch is wider than the lower, and that the upper teeth all around should overlap the lowers. I hardly agree with those who say we should imitate the natural arrangement with our artificial teeth in this respect, for if we observe the great majority of edentulous arches we will note that the anterior portion of the upper arch describes a wider segment than the lower, while as the jaws extend to the rear reverse is the rule. While I believe it proper to have the upper anterior teeth extend beyond the lowers and slightly overlap them, I think as we get to the bicuspids the buccal surfaces of both uppers and lowers should be on the same vertical plane, then as we get to the first molars above, they should be placed slightly inside of the buccal edges of the inferior molars, and the second molars above should then drop into a position where their bucco-occlusal edges would strike about midway of the buccal surface of the second lower molars.

By this arrangement of the arches, the upper should describe a wider circle anteriorly, and a smaller one posteriorly than the lower. I know that Dr. Bonhill advocated the arrangement of the bicuspids and molars in almost a straight line back from the cuspids, but that in many cases would land the last molar, especially in the upper, too far out into the cheek, and cause instability of the denture.—E. M. Kettig, in *Digest.*

**Training Children for Patients.** If the child of to-day be *well* trained in the value and care of the contents of its oral cavity, blessed is the dentist who may have the care of that child through its childhood and maturity; but if the child be badly trained, or not at all in these matters, woe to both patient and practitioner in the days to come.

"*Act well your part.*" If every dentist were to carry this out in the spirit as well as the letter, instructing each patient who is a parent, pointing out what is best for themselves as also for the little beings entrusted to their love and care, emphasizing what might seem trivial matters—for too often the trivial matters form the keynotes in the grand chorus of success; and Michael Angelo knew whereof he spoke when he said, "Perfection is made up of trifles and perfection is no trifle." If the parents *act well their part* toward the little ones, guiding the tiny hand till it becomes expert in the use of the tooth-brush, carefully scanning the teeth for the slightest evidence of decay, zealously guarding the child from outrageous tales of ill-treatment and cause of fear at the hands of the dentist—if the dentist himself acts toward the child the part of a gentleman in the old English sense of the word, in all respects, doing to the little one just as he would have done to himself under like circumstances—think you there would be septic mouths presented so often and a race seventy-five percent of whom dread the dental chair to such a degree that the mere anticipation of dental operations causes suffering?—Dr. Bessie Bennett in *Am. Journal of Dental Science.*

**Taking the Bite.** We know that the mandible is apt to protrude, and, in a less degree, move sideways. My method makes either of these mismovements more difficult to the patient and if correctly done it renders the protrusion impossible. It consists in the use of a piece of silk cord, about two feet long and 1-12 inch thick, so wound as to be devoid of stretch. This cord is passed around from the back of the neck, where it is tied in one fold, and then its ends are placed in the hands of the patient, who is directed to pull on them very forcibly at the time the bite is taken. Of course, it hurts a little, but it is intended that it shall do so, this also being the reason why the cord is no thicker. The patient is made to sit upright, with the head thrown back, and usually the precaution is taken of making a preliminary closure, directing the patient to pull very strongly on the cord, before taking the decisive bite. If he is apparently not pulling very hard, insist on his doing so until it hurts. When satisfied that he is making an honest effort, proceed to take the bite. If the method you employ for doing this is that which is known as the "squash" or "mush" bite, the wax must, of course, be left out of the mouth during the preliminary closures. When this simple operation is thoroughly performed it renders it impossible any protrusion of the mandible. I have given twenty-five years of study to the subject of the articulating impression, and was, I believe, the first to publish the method which has now become so well known through the true-bite plates; now, however, I give the palm to the little silk cord.—S. J. SPENCE.

**Peroxid Dentifrice.** Of late years special efforts have been made in the production of dentifrice compounds to bring together such ingredients as would upon application to the teeth and gums produce a sense of coolness combined of course with real antiseptic power. The acid dentifrice of the French Codex is a good example of this class of compounds, consisting, as it does, of a mixture of cream of tartar and sugar of milk, highly flavored with oil of peppermint. One ingenious investigator hit upon potassium chlorate as a valuable addition to tooth-powders, being evidently of the opinion that the salt would readily part with its oxygen when brought into contact with the moisture of the mouth. The antiseptic property of potassium chlorate applied in this way is more or less chimerical, and powders of this kind were never popular, what the laity demanded in a tooth-powder being a combination of substances and flavors that leave a pungent and slightly-sweetish taste in the mouth.

The application of the medicinal peroxids as dentifrice compounds is of particular interest to pharmacists, the subject having attracted a great deal of attention since Prof. Frankel's address to the Fifth International Congress of Applied Chemistry in explanation of the chemical and therapeutical applications of the newly-discovered peroxids of the alkali earths. Mr. Gane's application of this discovery to the manufacture of dentifrice compounds clearly marks a new era in the history of dentistry. He uses a calcium peroxid which in contact with the moisture of the mouth is resolved into milk of lime, hydrogen dioxid and water, the dioxid being in turn split up into its elementary constituents, thus liberating free oxygen at desired points, and securing at the same time an antacid action from the milk of lime.—Mr. Keenan in *Cosmos*.

**Local Analgesia With Stovain.** The eminent physiologist, Dr. Billon, has recently shown that "stovain," the new local anesthetic discovered by Mons. Fourneau, the distinguished French chemist, is twice less toxic than cocain. Since October, 1903, Professor Reclus has been testing the effects of this new agent upon man, and after nine months of continuous observation ventures to publish the report under review. He has found that the anesthetic power of stovain is identical with that of cocain, and that when carefully injected both agents have the same power of abolishing sensation.

Regarding the toxic action of stovain as compared with that of cocain, the author states that at the beginning of his investigations with cocain, in 1886, he found that five percent solutions of that drug would occasionally give rise to serious systemic disturbances, and that since using solutions of two to one and one-half percent, these physiological disturbances have been sensibly decreased. With stovain he has not observed any disagreeable after-effects, and believes with Dr. Billon that when injected in man stovain is less toxic than cocain. It is perfectly safe to hypodermically inject stovain even in greater quantities than cocain. Under its influence, the author has been able to perform a number of serious and long operations, such as the removal of large-sized tumors and the reduction of umbilical hernias.

Stovain is a vaso-dilator instead of a vaso-constrictor as is cocain. This presents both advantages and disadvantages. One of the inconveniences is that the field of operation is kept constantly filled with blood, but this slight disadvantage on the part of stovain is more than compensated by the effect of the agent upon the blood-vessels supplying the brain. When operating under cocain, it is the invariable rule to keep the patient in the horizontal decubitus, as it is impossible to operate with the patient in the sitting position. With stovain it will be possible to operate with the patient in the chair, thus greatly facilitating surgical work in the mouth and head.—Prof. Paul Reclus, in *Revue Odontologique.—Cosmos.*

**Cleaning the Teeth.** We all like to work in clean mouths. There is nothing that appeals to me more forcibly than the clean mouths that I frequently meet with in my practice, and I never lose an opportunity when I find them to compliment patients on the effort they have made to keep them clean. It has been a rule with me, before beginning any operations of filling, to put the mouth in a healthy and clean condition. I do this for two reasons: first, because it is more agreeable to work in a clean mouth, and the other to impress the patient with the importance of keeping the mouth clean. And in this connection I

am reminded of what Dr. Waters, of Boston, said years ago in the National Dental Association, when we were discussing the care of children's teeth. On the previous day he had had an experience, he said, that made him feel good. As he was walking up and down the platform at the station at Lowell waiting for the train, a fine-looking man of about forty years of age walked up to him, extending his hand, said, "I believe this is Dr. Waters, of Boston." He said, "Yes, but I do not recognize you." He said, "No wonder; you have not seen me for twenty-five years. When a boy my mother sent me to you to have my mouth attended to. You looked into it and said, 'Young man, I don't want to put my fingers in your mouth; it is too filthy. If you will go home and brush your teeth for two or three weeks, and then come back, I will look at your teeth.' I was so mortified that after I did get my teeth fairly cleaned I went to another dentist to have them attended to." And, opening his mouth, he said, "Did you ever see a cleaner set of teeth than that?" "Yes," I said, "they are clean." He insisted that I examine them carefully, and repeated, "Did you ever see a cleaner set than I am showing you?" I said, "Yes, they are certainly very clean, indeed." "I am indebted to you," he said, "for the lesson you taught me." When I see children careless about keeping the teeth clean—too busy to do it, as they say—I tell them this story, and often good results follow. It has been my practice with new patients to make them thoroughly cleanse the mouth before I attempt any operation in filling. Perhaps I do not do it as thoroughly as Dr. Smith, but I go over the teeth as carefully as I know how. When I am through, the patient is impressed with the importance of a clean mouth.—Dr. E. T. Darby in *Cosmos*.

**Death Under Gas Anesthesia.** Dr. Graham, of Ottawa, very kindly gives the following facts in connection with the death of a patient while teeth were being extracted, after the administration of nitrous oxide gas. Though deaths are very few under gas, yet they do occur, and we wish here to call attention to the necessity of being prepared for such accidents. Drs. Graham and Beattie were fully prepared, and nothing was neglected that might have been of assistance. There are dental offices not so well equipped to meet such emergencies.

"The patient came to the office about 4 p. m., on September 21st, accompanied by her mother and a friend, desiring to take laughing gas to have some teeth extracted. When I examined the mouth I found all the teeth in a badly-broken down condition, nearly all roots and diseased. I advised her to consult a physician and have choloroform administered. Her mother would not, under any condition, have her take chloroform, and said, 'Give her gas, and take out three or four that are troubling her most, because she is not very strong." She said she had eaten no breakfast or dinner, and was not the least nervous. I had our lady assistant remove her collar and belt and loosen up all tight bands, including her corsets. Dr. Beattie turned out six gallons of gas, which she inhaled without a struggle, and her respirations were deep and loud, pulse seemed weak. I ceased giving the gas when she had about five and a half gallons, and she was breathing nicely and seemed to be well anesthetized. I removed two teeth, and was about to extract the third when I noticed her change color, her lips turning to a deep purple. I immediately used the tongue forceps, turned back the chair as low as possible. Dr. Beattie injected one-thirtieth of a grain of strychnia into her arm, also put liquor ammonia fort. to her nose. We have two physicians in our building, and both were on hand in less than two minutes, but respiration and heart's action, I am convinced, had ceased from the moment I went to remove the third tooth. She never moved a muscle or made a sound. The doctors injected brandy, used amyl. nitrite and the electric battery (artificial respiration for one hour). When I told her friend, who remained in the room all the time, that she was in a dangerous condition, she said, 'Well, don't tell her mother, because she has heart trouble.' Her father told me afterward that she had always enjoyed good health, except when she was thirteen years of age, when the glands of her neck became enlarged and discharged freely. Her neck was scarred from ear to ear, but it seemed to be perfectly healed, as five years had elapsed since she had the trouble in her neck. We used artificial respiration from the time we noticed the change in color. There was no obstruction in the larynx, no vomiting or struggling, and not more than five and one-half gallons of gas were given."—*Dom. Journal.*

**The Combined Ray and its Application to Dentistry.** Dr. C. E. Rogers, of Seattle, has been investigating and perfecting this treatment for the last four or five years, which have resulted in the adoption of a three hundred candle-power incandescent light surrounded by a peculiar reflector which condenses the rays—not to a minute point, as a parabolic shade would do, but the condensation is of a larger range, which produces a new ray, called by him the "Combined Ray." He applies this term because of using the other rays of the light spectrum in conjunction with it, believing them to be of assistance in the efficiency of this new ray. It has the property of penetrating bone with as much facility as soft tissue. As an illustration, suppose a bullet to have lodged in one of the large bones of the body, the femur, for instance; a photograph with this light would show the bullet, but not the bone, while with the X-ray the bullet would be invisible, but the bone, with the ragged entrance, would be seen. It has great penetrating force, and there seems to be scarcely any danger from its use, as the burn which is possible from the extreme heat is prevented by indications from the patient before it comes to that point. This is quite different from the X-ray; therefore it can be used without danger. In its application to disease it has a large field, including many of the conditions met by the dentist, and in a large majority of these diseases the results are almost miraculous.

In all forms of inflammation and pus formation it seems to be especially applicable. My attention was attracted by this feature of the light. Therefore I had one installed in my office, and the results produced by its use have given me the greatest satisfaction.

My first application was made in the case of a patient who came to me having a severe headache. In about seven minutes I was able to relieve the pain entirely, placing her in a grateful frame of mind and in a much better condition to stand the necessary pain accompanying a dental operation. I have as yet to find the headache which cannot be controlled by this light in from seven to ten minutes. My next application was in the case of a patient having acute neuralgia, indicating itself by the usual paroxysmal pain in the supra and infra-orbital branches of the fifth nerve. From seven to ten minutes' application of the light was all that was required to entirely relieve the pain. I then had occasion to apply the light to myself, having been afflicted for years with acute attacks of neuralgia, occurring possibly three or four times a year, always in the supra and infra-orbital branches. It is usually so severe that I am compelled to drop my work, sometimes in the middle of an operation. From ten to fifteen minutes' application of the light relieved me entirely, and I have not had a return since.

I am using it in the treatment of pyorrhea after having thoroughly cleansed the roots, and hope to see beneficial results. The time since the treatment has not been sufficient to allow me to speak definitely, but the indications are very favorable.

While the cases above mentioned should receive our attention and can be controlled with the light, the most important field lies in the almost miraculous relief given to patients suffering from a suppurating tooth. I believe that from one to three applications, of ten to twenty minutes each, will give entire relief to practically every case of this class. It destroys the pus-forming germ, and the pain from pressure disappears almost immediately, allowing the operator to open and treat the tooth without further inconvenience to the patient. I have had cases come to a head in from two to three hours after the first application; when opened there would be only a drop or two of pus. It has given immediate relief in every case of suppuration on which I have used it, and in a number of cases, I am sure, it has saved the patient from twenty-four to forty-eight hours' suffering. To better illustrate the usefulness of this light, I will cite the case of a patient who was brought to my office, having ten days previously had a lower wisdom tooth removed, the dentist using a local anesthetic. Shortly after the operation her face began to swell and the muscles stiffen, until she was unable to take nourishment except through a straw. A consultation of the dentist and a physician resulted in a decision that the condition was caused by her having taken cold in the wound. The swelling had now involved the glands of the neck and was very sensitive to palpitation. It was with difficulty that I could place my mouth mirror in a horizontal position between her teeth. Twenty minutes' application of the light removed all the pain; it was scarcely sensitive to touch and she was able to open her mouth nearly three-quarters of an inch, and on her arrival home she took solid food for the first time in four days. Two applications of the light cured the case.

After this and many more marvelous results I feel warranted in stating that this light is surely a boon to suffering humanity, and I am sure that its application would be universal if the results of my experience could be realized by others. The relief of pain is a part of our work, and nothing has ever come to my notice which, in the same length of time, insures such wonderful results.—Dr. A. E. Peck, in *Dental Summary*.

